# Cross-sex hormone treatment and own-body perception: behavioral and brain connectivity profiles

**DOI:** 10.1038/s41598-020-80687-2

**Published:** 2021-02-02

**Authors:** Behzad S. Khorashad, Amirhossein Manzouri, Jamie D. Feusner, Ivanka Savic

**Affiliations:** 1 Department of Women’s and Children’s Health, Karolinska Hospital, Karolinska Institutet, Q2:07, 171 76 Stockholm, Sweden; 2 Department of Psychology, Stockholm University, Stockholm, Sweden; 3 Semel Institute for Neuroscience and Human Behavior, University of California Los Angeles, Los Angeles, USA; 4 Department of Neurology, University of California Los Angeles, Los Angeles, USA

**Keywords:** Neuroscience, Cognitive neuroscience, Neural circuits, Social neuroscience

## Abstract

Referrals for gender dysphoria (GD), characterized by a distressful incongruence between gender identity and at-birth assigned sex, are steadily increasing. The underlying neurobiology, and the mechanisms of the often-beneficial cross-sex hormone treatment are unknown. Here, we test hypothesis that own body perception networks (incorporated in the default mode network—DMN, and partly in the salience network—SN), are different in trans-compared with cis-gender persons. We also investigate whether these networks change with cross-sex hormone treatment. Forty transmen (TrM) and 25 transwomen (TrW) were scanned before and after cross-sex hormone institution. We used our own developed Body Morph test (BM), to assess the perception of own body as self. Fifteen cisgender persons were controls. Within and between-group differences in functional connectivity were calculated using independent components analysis within the DMN, SN, and motor network (a control network). Pretreatment, TrM and TrW scored lower “self” on the BM test than controls. Their functional connections were weaker in the anterior cingulate-, mesial prefrontal-cortex (mPFC), precuneus, the left angular gyrus, and superior parietal cortex of the DMN, and ACC in the SN “Self” identification and connectivity in the mPFC in both TrM and TrW increased from scan 1 to 2, and at scan 2 no group differences remained. The neurobiological underpinnings of GD seem subserved by cerebral structures composing major parts of the DMN.

## Introduction

Gender dysphoria (GD) is capturing increasing interest in neuroscience, yet the underlying mechanisms have not been fully characterized. Individuals who are transgender identify with the gender opposite to their at-birth assigned gender^1,2^, whereas cisgender refers to those who identify congruently with their at-birth assigned gender. Many individuals who are transgender, experience GD defined as distress due to incongruence between their experienced gender and their body sex phenotype, and can be diagnosed with Gender Dysphoria (in DSM-5). The most frequent treatment for GD is cross-hormone therapy, which in most cases results in reduction of dysphoria. The neurobiological mechanisms of the perceived alleviation of dysphoria are not clear, nor is the neurobiology of GD. For many years, GD has been viewed to be a result of altered cerebral sexual differentiation^3^. However, while imaging studies have been mostly consistent in reporting structural and functional differences between cisgender males and females, showing larger relative caudate and hippocampus volume in women, larger putamen and amygdala volume in men, greater cortical thickness in women, stronger functional connectivity in the posterior cingulate cortex in women, and greater fractional anisotropy (indexing white matter integrity) in men^4–12^, findings from the brain imaging studies on GD in the last two decades have been much less consistent. With respect to TrM there are studies reporting a cerebral pattern congruent with that of cisgender females^13,14,19,26^, whereas other studies describe partly similar structural and functional neural characteristics as in cisgender males^15,16^. Still, other reports describe a pattern different from both cisgender male and cisgender female control groups^17–19,26^. Corresponding data from studies of TrW appear more consistent, e.g., a “female features” have been reported both with respect to fractional anisotropy (FA), (reflecting white matter connections), as well as with respect to cortical thickness^20,21^. Some findings, however, diverge from such ‘female features’, showing values in between those of male and female controls regarding white matter integrity^22^ and a thicker mesial frontal lobe cortex compared to cisgender male controls though male and female cisgender controls did not differ in this region^23^. Thus, while it seems that cerebral morphology and function differ between cisgender and transgender subjects, there has not been consistent evidence for systematic features^13–15,23–27^.

As we discussed in papers elsewhere^16,19,28^, a conceptually important reason for the lack of clarity regarding the tentative cerebral underpinnings of GD is that most reports do not address *the principal feature of this condition*—a strong perception of incongruence between one’s sense of self and one’s body, a discomfort with one’s own body, and a feeling of estrangement towards one’s physical sex^29^. This incongruence may well be unrelated to cerebral sex dimorphism. Results from a series of structural and functional MRI studies combined with behavioral data^16,25,28^ led us to recently propose a hypothesis that GD is characterized by a functional disconnection between systems in the brain that process the perception of self (“self-referential”), and those that mediate own-body perception.

Several research groups, using partly different methodologies, have reported that self-referential processing is mediated by a specific network in the brain encompassing portions of the occipito-parietal (the extra-striatal body area [EBA], the fusiform body area [FBA], the temporoparietal junction [TPJ]), the mesial prefrontal cortex [mPFC], and the anterior cingulate cortex [ACC]^30–32^. This network largely overlaps with the default mode network (DMN), (the mPFC, precuneus, bilateral parietal cortices), which is known to be active during rest and when letting the mind wander^33–35^. In addition, it incorporates parts of the salience network (SN, consisting of the fronto-insular and ACC). Investigating relatively large cohorts of transgender individuals with GD, we found significant functional and structural differences between transgender and cisgender persons in portions of these networks. Firstly, the cortical thickness (Cth) in both TrM (transmen, male to female transgender) and TrW (transwomen, male to female transgender) was greater in the mPFC and bilaterally in the parieto-occipital cortex^25^ compared with cisgender controls. Secondly, the diffusion properties of white matter connections between these areas were different in TrM as well as TrW compared with cisgender controls^16^. Thirdly, functional connections within the ventromedial PFC and pACC of the DMN were weaker among TrM compared to cisgender controls^25,28^. Together, these data confirm that own-body processing circuits incorporated in the DMN and the salience network are indeed involved in GD. These results also raise several new questions: (1) are functional connections within the DMN affected among TrW similarly to TrM? (2) are these circuits targeted by cross-sex hormone treatment among transgender populations in general? (3) given that cross-sex hormone treatment is reportedly beneficial—improving congruence between gender identity and own-body, and reducing dysphoria, are the effects of this treatment related to possible changes in own-body processing as measured with the Body Morph (BM) test [for a description of the BM test, see^28^]. In addition, sex hormones (testosterone and estrogen) have been reported to have differential effects on cerebral functional connections^36^. It has been reported that higher levels of estrogen are linked to increased functional connectivity^37^ and administration of estrogen to women and rats increases amygdala–PFC connectivity^38^. Furthermore, these connections are found to be stronger in women with higher estrogen levels^39^. On the other hand, high endogenous testosterone levels attenuate resting-state amygdala-PFC coupling in adolescents^40^. These data from cisgender controls led us to wonder whether similar effects apply to transgender populations with GD. To address these issues, we carried out longitudinal MRI measurements of functional resting state connectivity before and after the start of cross-sex hormone treatment in 40 TrM, 25 TrW with GD, and in 15 cisgender controls (not receiving cross-sex hormones).

We hypothesized that if cerebral changes are found specifically in the networks involved in own-body processing and in both TrW and TrM, then these changes may be related to the process of self-own body congruence. We expected to detect pre- to post-treatment changes in cerebral connectivity in the DMN. We chose to study DMN because it is shown to be robust with high test–retest reproducibility, and because it has in several previous studies (including our own) shown to overlap with the brain areas involved in the perception of own body in the context of self (e.g., see^25,28,30,41^). We assumed that tentative between-scan changes in functional connectivity changes would be associated with the altered degree of own body—self-perception (as measured by the 0% morph ratings).

## Materials and methods

### Participants

For a detailed description of the methods please see our previous publications^21–23^. Forty TrM with GD (Mean age = 22.93 ± 5.28 age range = 18–40 years old; education 13.38 ± 1.95, range 9–17.5 years), 25 TrW with GD (Mean age = 27.96 ± 7.12; age range = 20–50 years old; education 14.6 ± 2.46, range 8–19 years), 5 cis gender women (Mean age = 30.6 ± 8.849; age range = 20−44 years old; education 15.3 ± 2.11, range 13–18.5 years) and 10 cis gender men (Mean age = 30.30 ± 7.40, age range = 20–42; education 17.35 ± 4.11 years; range 13–24) participated in the longitudinal study of possible treatment effects. All participants were tested for handedness according to Oldfield^42^.

Transgender participants were recruited at The Center for Andrology, Sexual Medicine and Trans medicine (ANOVA), the integrated center for Transgender Medicine, Karolinska University Hospital (Stockholm, Sweden), from the 1st of January, 2011 to the end of November, 2018. Participants aged between 18 and 45 years who, after diagnostic evaluation, fulfilled the criteria of binary GD according to The International Classification of Diseases (ICD) version 10, were invited to participate in the study. None of the participants had received hormone treatment at the time of scan session 1, or gender confirmation surgery at the time of scan session one or two. Individuals on current hormonal treatment, known chromosomal or hormonal disorder, or current psychiatric disorder—as confirmed by the Mini International Neuropsychiatric Interview [MINI]^43^,—including body dysmorphic disorder, neurological or other major medical disorders, or any current use of medications with psychotropic effects (antipsychotic or antiepileptic agents, lithium, benzodiazepines or opioid analgesics) were not included. All participants with GD received cross-sex hormone treatment after the first scan, and none of them had affirmative surgery. We also excluded persons who had either diagnosed autism spectrum disorder (ASD) (before being referred to the team) or had shown clinical signs of ASD when being assessed by the team.

Exclusion criteria for the cisgender group were neurological or psychiatric disorders, substance use disorders, family history of psychiatric disorders, and ongoing medication. Moreover, if they experienced any major diseases, life trauma, or initiated ongoing medication (other than cross-sex hormones) between the 2 scans, they were excluded from the study. All cis-women participants had regular menstrual cycles and were investigated during the second week after their menstruation. None was treated with contraceptives. All subjects were asked about possible endocrine problems before inclusions, and none were reported. For demographical details, please see Table 1.

**Table 1:** Demographics.

	Transmen (n = 40)			Transwomen (n = 25)			Controls (n = 15)		*F* value		*p* value
	n	Mean (SD)	Range	n	Mean (SD)	Range	n	Mean (SD)	Range		
Age in years	40	22.93 (5.28)	18–40	25	27.96 (7.11)	20–50	15	30.50 (7.60)	20–44	9.40	< 0.0001
Years of education	38	13.38 (1.95)	9–17.5	24	14.6 (2.46)	8–19	15	16.70 (3.60)	13–24	9.40	< 0.0001
Handedness	25	73.8 (29.0)	− 20 to 100	18	70.0 (30.0)	20–100	15	70.80 (32.60)	− 5.89 to 100	1.90	NS

Handedness has been assessed according to Oldfield^42^.

F-values from group comparisons (one-way ANOVA). Transmen were significantly younger than both transwomen and cisgender controls. Both transmen and transwomen had fewer years of education than cisgender controls. No difference in handedness were found. NS-not significant.

The study was approved by the ethical committee of the Karolinska Institute (application number: Dnr 2011/281-31/4) considering its compliance with the Committee’s ethical standards which follow the 1964 Helsinki declaration and its later amendments. Informed consents were obtained from all individual participants included in the study prior to participation.

### Procedure

All groups were scanned twice using MRI: TrM before starting testosterone treatment and TrW before starting antiandrogen (Androkur) and estrogen treatment. The majority of patients participated in a previous study^44^, in which we reported the sex hormone treatment, plasma level assessment and changes in blood hormone levels more in detail. Briefly, in TrM and TrW, serum hormone levels were assessed by routine clinical check-ups and the assessments closest in time to the MRI sessions were used for the purpose of this study.

Hormonal treatment in the transgender participants as well as their hormonal analysis pre- and post-treatment have been described in more detail elsewhere^44^. No blood samples were collected in cisgender controls.

TrM and TrW were scanned again at least 6 months after sex hormone initiation (TrM: average interscan interval 15.84 ± 6 months, range 6 to 27 months; TrW: 14.28 ± 8.76 months, range 7 to 48 months). Cisgender controls were investigated before, and after a period of time without intervention (average interscan interval 34.8 ± 6 months, range 27.6–38.4). There was a significant difference in the period between two scans between transgender and cisgender participants with the latter having a longer interval (unpaired t-test, *t* (78) = 10.067, *p* < 0.0001).

### Own body perception test

To explore behavioral responses to the perception of the own body, we carried out a “body perception test,” in which participants, outside the scanner, viewed photographs of their bodies morphed by 20% increments toward either cis male or cis female bodies [for details of the procedure, see^28^], In sum, each participant’s picture was morphed towards those of three different female and three different male target pictures at degree intervals of 20%, using FantaMorph Software, version 5.0 (Abrosoft http://www.fantamorph.com/). Eleven morph conditions resulted, ranging between − 100% opposite to sex assigned at birth to + 100% towards sex assigned at birth. “100%” refers unmorphed bodies of a completely different individual, while 0% refers to the original unmorphed image of the participant. For each image presented the test person was asked to respond “to what degree is this picture you?” This allowed us to obtain ratings that index own body identification with images of their unmorphed body (0% morph images), as well as their body morphed to appear more masculine or more feminine (Body morph index).

During each experimental trial, the 60 morphed and one unmorphed images presented on the computer screen for either 0.5 or 2 s (short vs. long exposure times), randomly with respect to both morph percentage and exposure times. Participants were instructed to press computer keys 1–4 (1 corresponding to 0–25% me, 2 to 25–50% me, 3 to 50–75% me, and 4 to 75–100% me). In addition to obtaining 0% morph ratings, we calculated an “own body perception index” by multiplying each degree^1–4^ of “self” rated for each morph with the degree of each morph. Positive values (arbitrarily) indicated morphs to their sex assigned at birth (0.20, 0.40, 0.60, 0.80, and 1.00) and negative values for morphs to the sex congruent with their gender identity, and opposite assigned sex at birth (− 0.20, − 0.40, − 0.60, − 0.80, and − 1.00). These weighted values were averaged for each participant across ratings for all 61 images and then divided by the number of rated images, providing an average index of self-perception for each participant. Thirty-seven of the 40 TrM and fifteen of the 25TrW performed the body perception test at both time points, and only 5 controls performed the test twice (due to the fact that this part of the experiment was added after MRI data collection had begun). The data of the body perception test were not compared with the cisgender controls for each gender separately, as in our previous studies we did not detect any sex differences in the body perception index^28^, independently of gender identity (cis or trans gender)^41^.

### Data acquisition

This acquisition of MRI data has been detailed elsewhere^25^ and will be reviewed here too. The images were acquired on a 3-T MRI scanner (Discovery 3 T GE-MR750, General Electric) equipped with a 32-channel phased array receiving coil for all the sequences. 3D T1-weighted Spoiled Gradient Echo pulse sequence (SPGR) images were acquired with 1 mm 3 isotropic voxel size (time echo [TE] = 3.1 ms, time repetition [TR] = 7.9 ms, time to inversion [TI] = 450 ms, field of view = 24 cm, 176 axial slices, flip angle 12°). Resting-state functional MRI (fMRI) was performed with a gradient echo pulse sequence using a voxel size of 2.25 × 2.25 × 3 mm (TE = 30 ms, TR = 2500 ms, FoV = 28.8 cm, 45 bottom-up interleaved axial slices, 3 mm thickness, flip angle 90°). During the fMRI session, which lasted 8 min, participants were instructed to close their eyes, not to try to solve any special task but just “let the mind wander,” and to try not to fall asleep.

### Data analysis: resting state fMRI and calculation of functional connectivity

Preprocessing of the functional images was performed using SPM8 (Welcome Department of Cognitive Neurology) according to the standardized procedure including field map correction using B0 images during the warping procedure (applying VDM’ option in the FieldMap Toolbox), for detailed description, please see^25^.

Motion correction was conducted with 18 movement regressors (six linear, their squares and cubes, SPM8 software). The spatial parameters were then applied to the slice-timed and realigned functional volumes that were finally resampled to 2 × 2 × 2 mm voxels and smoothed with a 6-mm full-width at half-maximum kernel.

The data were then analyzed in FSL software v5.0 (FMRIB Software Library, Oxford, http://fsl.fmrib.ox.ac.uk/), using a high-pass filter at 100 s before running individual independent component analyses (ICAs)^45^, using Multivariate Exploratory Linear Decomposition into Independent Components (MELODIC), Version 3.14, with automatic determination of dimensionality. The resulting component maps were manually classified into components of interest and nuisance components (including white matter and CSF) in accordance with the criteria proposed by Kelly^46^. The nuisance components were subsequently regressed out of the original dataset using fsl_regfilt.

**Figure 1:**
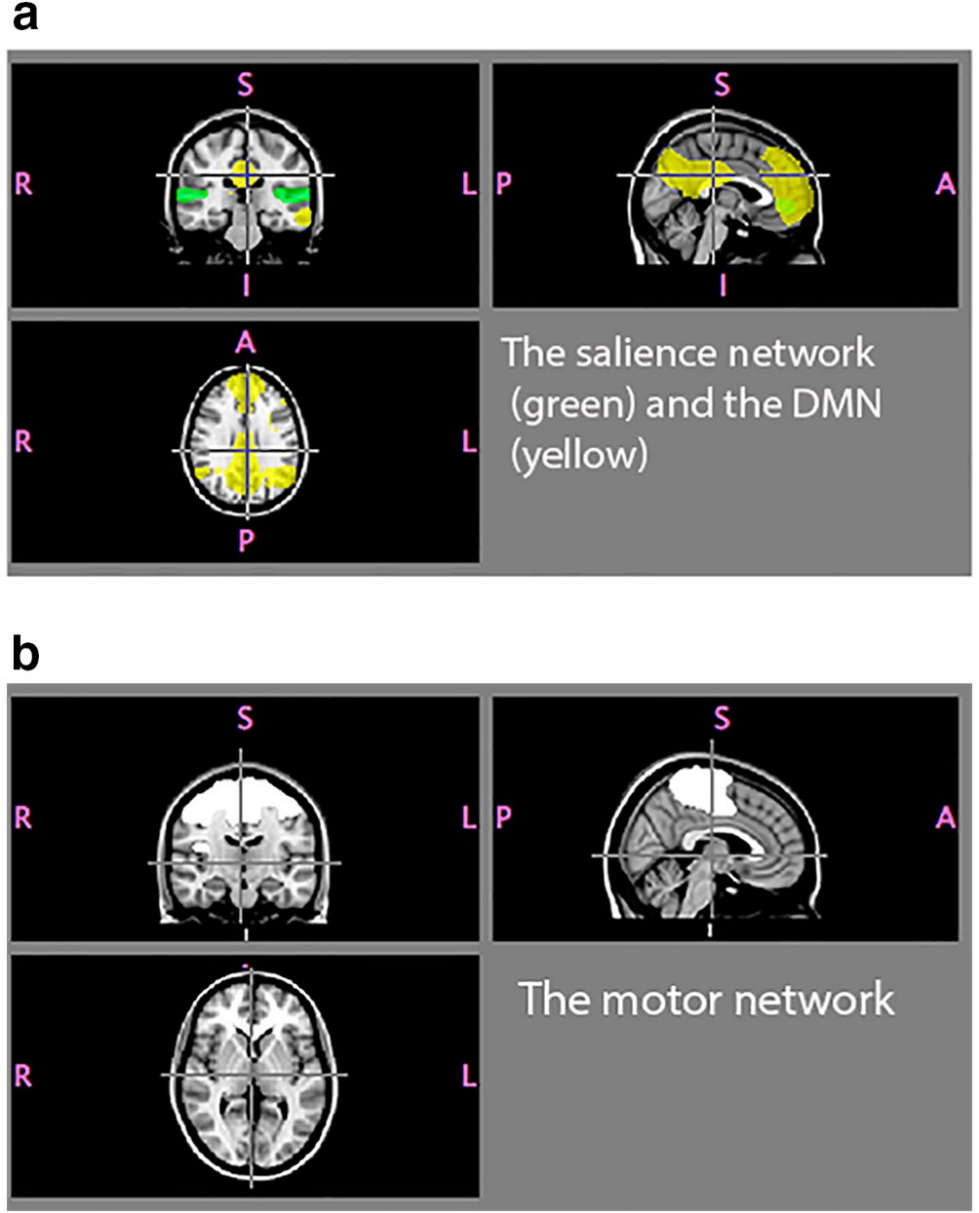
The networks investigated. (**a**) Illustration of the DMN (yellow) and salience (green) networks superimposed on the MNI standard brain. (**b**) The motor network is indicated in white.

Group concat-ICA was performed on the entire cleaned dataset, resulting in 20 components. These components were used to perform dual regression analysis, with the resulting general linear model (GLM) parameter estimate images fed into FSL’s Randomise tool for non-parametric permutation inference^46,47^ in order to test our hypothesis about changes in connectivity pre- to post-treatment, and to test for differences among groups. In light of our a priori hypothesis, we specifically examined the DMN, assuming that major pre- to post-treatment effects (our primary hypothesis) would be observed in this network. We also examined the salience network, since we previously found associations in this network in cisgender controls between BM index and right insula connectivity^28^. As a reference “control” network—not expected to show changes in functional connections due to treatment—we also examined the motor network. Other components were not used in the analysis for the present publication. The coverage of the three networks as used is shown in Fig. 1. The statistical design included using age and mean DVARS (Root Mean Square intensity difference of volume N to volume N + 1^47^ as a nuisance covariate. DVARS represent an index of the effects of motion on image signal intensity.

To assess possible baseline (before hormone treatment) differences between TrM and controls and TrW and controls, dual regression was run between groups using *scan 1 images*, with age and years of education as covariates of no interest. The significance threshold was set at p < 0.017 (0.05/3), FWE corrected, to account for independent testing of three separate networks.

### Evaluation of the body morph test

Between (one-way ANOVA, Scheffe’s post hoc test, *p* < 0,05), and within group (paired t-test, p < 0.05) comparisons were carried out using 0% morph and BM index data in separate computations (group comparisons with repeated measure ANOVA were not applicable, as all the controls did not perform the BM test at two occasions). The unmorphed images were presented only once in all three groups, in order to avoid recognition effects. Furthermore, several participants did not respond when viewing their own unmorphed body. To maximize the number of data points, the between- and within-group comparisons for 0% morphed images were carried out using the responses for long and short image exposure in the same analysis (there was no significant difference between mean ratings of long and short exposures of the 0% morph images). Likewise, the control data consisted of combined responses from males and females. Because cisgender controls did not do the BM test at two occasions the post treatment comparison was carried out using their data from the first visit. Please note that the unmorphed images in body morph test before and during treatment were different as separate photos for the 0% morph condition were taken before, and during treatment (at the time of scan 1 and scan 2).

For the BM index, the number of data points was independent of whether there were non-responses for the 0% images, and we calculated both long and short view responses separately, as shown in Table 2. However, for the sake of consistency, and as their mean responses for long and short view responses were not significantly different, the statistical comparisons between transgender and cis gender populations were carried our pooling the short and long view data as described for the 0% ratings.

**Table 2:** Results from the BM test.

	0% morph images pre-treatment	0% morph images post-treatment	BM index pre-treatment, 0.5 s exposure	BM index pre-treatment, 2 s exposure	BM index post-treatment, 0.5 s exposure	BM index post-treatment, 2 s exposure
Cis controls (n = 15)	2.9 ± 1.1		26.0 ± 15.5	29.3 ± 18.0		
TrM (n = 40)	1.96 ± 1.0	2.8 ± 1.0	− 14.9 ± 32.8	− 6–5 ± 63.3	− 21.3 ± 21.1	− 24.5 ± 49.9
TrW (n = 25)	1.85 ± 1.0	2.4 ± 1.0	− 13.8 ± 29.5	− 8.8 ± 57.6	− 21.2 ± 24.1	− 22.6 ± 45.7

Ratings for 0% morphed (unaltered) images ranged from 0 (not me at all) to 4 (entirely me). Positive values on the BM index indicate greater self-identification with their body morphed to other bodies that are the same as their birth assigned sex (as seen in cis-controls); negative values indicate greater self-identification with their body morphed to other bodies that are the opposite to their birth assigned.

## Results

### Demographics

TrM were significantly younger compared to TrW and cisgender participants (Table 1). Testosterone levels increased with treatment in all TrM, and were significantly higher at Visit 2 than at Visit 1, whereas estrogen levels decreased and were significantly lower at Visit 2 compared to Visit 1 ([s‐testosterone, t = 5.0, *p* < 0.001; s‐estrogen t =  − 11.9, *p* < 0.001]). Likewise, testosterone levels decreased and estrogen levels increased in all TrW from Visit 1 to Visit 2 (s‐testosterone, t =  − 10.2, *p* < 0.001; s‐estrogen t =  − 5.6, p < 0.001). TrM were investigated, on average 10.4 ± 5.8 months after institution of testosterone treatment, and TrW 11.1 ± 7.8 months after institution of estrogen/anti‐androgen treatment.

### Body morph ratings

The mean responses in the respective group before and after treatment are presented in Table 2. Before treatment both TrM and TrW rated own unmorphed images significantly lower ‘as self’ than cis controls *F*(2,77) = 5.0, *p* = 0.01; Scheffe’s post hoc test disclosed that difference from controls was present in TrM (*p* = 0.031) as well as TrW (*p* = 0.018). Paired t-test showed significant increase in TrM in 0% morph ratings (pooling long and short exposure) with testosterone treatment (*p* = 0.010, n = 37, t = 2.7) and in TrW with estrogen treatment (*p* = 0.036, n = 16, t = 2.2). No group difference was found in 0% morph ratings at the time of second scan, *F*(2,65) = 2.37, *p* = 0.103.

One way ANOVA using BM index (long and short view merged) as a variable of interest showed significant overall group difference before the treatment [*F*(2,77) = 21.5, p < 0.001. This difference was present in relation to TrM as well as TrW (*p* < 0.001 for both comparisons), Scheffe’s post hoc test. No significant difference was detected between the two transgender groups (*p* = 0.952).

Although the BM index decreased numerically from test 1 to test 2 (indicating a greater congruence with the desired sex) there were not significant within group changes. This was due to an overall large variance in the output, before as well as after treatment. After treatment the BM index remained significantly different (and even more tuned to the desired sex) for pooled short and long view data [*F*(2,65) = 24.03, p < 0.001], both for TrW and TrM (*p* < 0.000 for both), again, without a significant difference between the two transgender groups (*p* = 0.846).

### Connectivity patterns within the DMN

**Figure 2:**
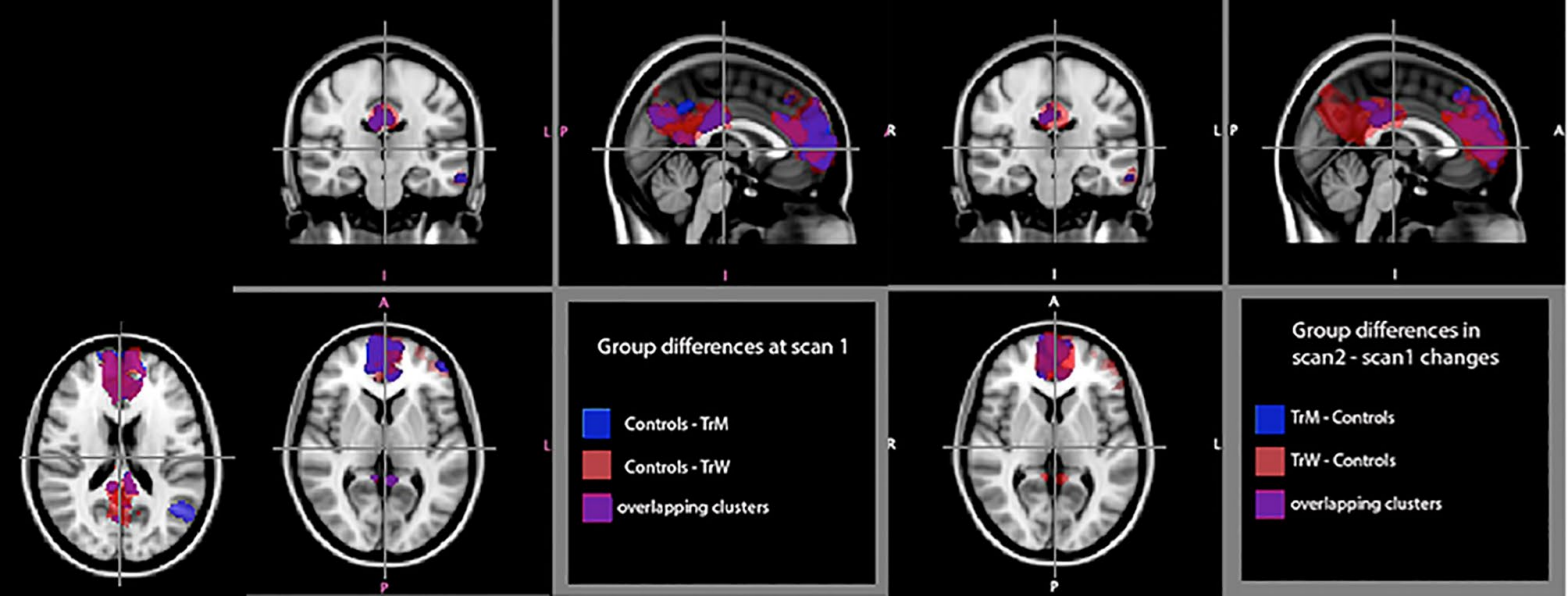
Significant group differences in rs-fMRI within the default mode network (DMN). Differences at scan 1 are shown to the left in the image. Group differences in scan2-scan1 changes are shown to the right in the image. Differences between controls and transmen are indicated in blue. Differences between controls and transwomen are indicated in red. Overlapping clusters are indicated in purple. All clusters are calculated at p < 0.0083, corrected (minimum cluster size 1.0 cc). MNI coordinates for the crosshair are 4, − 16, 2.

Compared to cisgender controls, at scan 1 both TrM and TrW showed significantly weaker connections within the DMN in the anterior cingulate cortex (ACC), mesial prefrontal cortex (mPFC), precuneus, the left angular gyrus and a portion of superior parietal cortex (Table 3, Fig. 2). There was no significant difference between the two transgender groups. Within-group comparisons at scan2–scan1 showed sub-significant increase in mPFC among TrW and TrM, and a significant decrease in mPFC among controls. In TrM, an additional increase in the precuneus was also observed. Notably, at scan 2, all significant group differences disappeared.

**Table 3:** Connectivity in DMN before (scan 1) and after (scan 2) cross-sex hormone treatment in TR persons, (no treatment in controls).

		Region	Cluster size (cc)	MAX	X	Y	Z
DMN	**Between-group comparisons, scan 1**						
	Controls > TrW	mPFC	8.9	1.0	8	52	− 14
		lPFC^a^	3.7	1.0	− 48	16	32
			2.7	1.0	− 48	42	− 2
		BA 32 (ACC)	1.8	1.0	6	38	18
		L orbitofrontal cortex, BA 10 (PFC)	1.2	1.0	− 38	50	− 12
		BA 23 (PCC), precuneus	5.6	0.99	− 4	− 46	2
		L BA39 (angular gyrus)^b^	2.9	0.99	− 42	− 62	16
		L parietal cortex	1.8	0.99	− 26	− 72	44
		R parietal cortex	1.0	0.99	48	− 56	32
	Controls > TrM	R BA 32 (ACC)	7.9	1.0	10	32	16
		L BA 8 (dorsal medial prefrontal cortex	1.7	0.98	− 16	48	40
		L PFC^a^	2.2	0.99	− 54	40	− 4
		Precuneus	3.9	1.0	− 6	− 38	20
		L Ba39 (angular gyrus), superior parietal cortex	1.8	0.99	− 48	− 60	16
	**Between-group comparisons, scan 2**						
		No significant clusters					
	**Within-group comparisons, scan 1 > scan 2 or the reverse**						
	Controls (scan 1 > scan 2)	R mPFC	2.1	0.99	6	58	− 8
	TrM (scan 2 > scan 1)	R PFC^c^	*0.7*	*0.98*	*6*	*62*	*24*
	TrW (scan 2 > scan 1)	R mPFC^c^	*0.98*	*0.9*	*2*	*60*	*16*
	**Between-group comparison, scan 2 > scan 1**						
	TrW > controls	mPFC	1.1	0.99	16	52	16
					4	38	− 8
	TrM > controls	mPFC	8.5	1.0	6	54	0
		Precuneus	1.9	1.0	10	− 30	28

DMN, default mode network; mPFC, Mesial prefrontal cortex; lPFC, Lateral prefrontal cortex; ACC, Anterior cingulate cortex.

Calculations at 1 − *p* > 0.98, cluster size > 1.0 cc; Italics indicate sub-significant clusters (1 − *p* < 0.98, cluster size < 1.0 cc).

The CON < TrM and TrW clusters in anterior DMN could be partly constituted by lower connectivity in scan 2 vs 1 among controls, in addition to the increased connectivity in TrM and TrW in scan 2 vs 1. Scan 2 > 1 in the posterior DMN are constituted by scan 2 increases among the transgender group.

^a^Includes part of the dorsolateral prefrontal cortex.

^b^Includes superior parietal cortex.

^c^Indicate sub-significant levels (cluster size < 1.0 cc), none of the other contrasts showed any clusters.

### Connectivity patterns within the salience network

No group differences were detected in the salience network at scan 1. There was, however, a significant scan2–scan1 connectivity increase in the mPFC (partly overlapping with a cluster in DMN), and in the insular cortex in both TrM and TrW, but not in controls. Group comparison for scan2–scan1 showed significantly increased connectivity in the mPFC/ACC among TrW compared to controls. No group differences were detected in the salience network at scan 2 (Table 4).

**Table 4:** Connectivity in salience network before (scan 1) and after (scan 2) cross-sex hormone treatment in TR persons, (no treatment in controls).

	**Between-group comparisons, scan 1**						
Salience network		No significant clusters					
	**Between-group comparisons, scan 2**						
		No significant clusters					
	**Within-group comparison, scan 1 > scan 2 or the reverse**						
		Region	Cluster size (cc)	MAX	X	Y	Z
	Controls (scan 1 > scan 2)	No cluster					
	TrM (scan2 > scan 1)	L insular + superior temporal cortex	1.5	0.99	− 64	− 12	− 2
		L mPFC	1.2	0.99	− 22	40	-8
	TrW (scan2 > scan 1)	L insular + superior temporal cortex	1.5	1.0	− 66	− 12	0
		L mPFC	1.4	1.0	− 22	38	− 8
	**Between-group comparisons, scan 2 > scan 1**						
	TrM > controls	No significant clusters					
	TrW > controls	R BA 10, mPFC	1.2	1.0	4	54	-6

mPFC, mesial prefrontal cortex; lPFC, lateral prefrontal cortex; ACC, anterior cingulate cortex.

Calculations at 1 − p > 0.98, cluster size > 1.0 cc.

### Connectivity patterns within the motor (reference) network

No significant between scan differences were detected in the functional connectivity in the motor network, neither within- nor between-groups. Functional connections were, however, greater in controls compared to both transgender groups (in the first, as well as the second scan) in the sensory motor cortex, along the body representation area of the homunculus (Table 5).

**Table 5:** Connectivity in motor network before (scan 1) and after (scan 2) cross-sex hormone treatment in TR persons, (controls were not treated).

	**Between-group comparisons, scan 1**						
Motor network	Con > TrM	R sensory motor cortex, (leg area)^a^	5.7	1.0	6	− 6	40
	Con > TrW	L sensory motor cortex, (body area)	13.4	1.0	− 36	− 26	38
	**Between-group comparisons, scan 1, and scan 2**						
	Con > TrM	L sensory motor cortex (leg area)^a^	7.9	1.0	12	− 18	38
	Con > TrW	L sensory motor cortex, (body area)	7.8	1.0	− 32	− 26	42
	**Within-group comparison, scan 1 > scan 2 or the reverse**						
	Controls (scan 1 > scan 2)	No significant clusters					
	TrM (scan2 > scan 1)	No significant clusters					
	TrW (scan2 > scan 1)	No significant clusters					
	**Between-group comparisons, scan 2 > scan 1**						
	TrM > controls	No significant clusters					
	TrW > controls	No significant clusters					

mPFC, mesial prefrontal cortex; lPFC, lateral prefrontal cortex; ACC, anterior cingulate cortex.

Calculations at 1 − p > 0.98, cluster size > 1.0 cc.

^a^Covers both right and left side.

### Covariation analysis: connectivity changes vs. own body perception changes in the DMN

As 0% morph rating showed an increase with treatment, we investigated if there was any association between an increase in own body perception in the combined TrM and TrW group and connectivity changes in the DMN (the network showing significant within and between-group changes related to treatment). We performed dual regression using participants’ changes in 0% morph ratings as a covariate and scan2 > scan1 contrast in the DMN as the connectivity dependent variable, followed by statistical testing using Randomise software. A significant cluster was detected in the left precuneus/posterior cingulate with a positive association between the change in 0% morph scores and change in connectivity with treatment, such that greater increases in ‘self’ ratings corresponded to greater increases in connectivity (cluster size = 1.3 cc; maximum 1 − p value = 0.987, [corresponds to *p* = 0.013]; local maximum x = − 18, y = − 58, z = 16).

## Discussion

In this study, we hypothesized that the neurobiology of GD is linked to cerebral networks encompassing self-referential and body ownership regions, primarily with the DMN. We recently found that this network is activated during own-body processing among cisgender, as well as transgender individuals^41^, but that the stimuli activating this distributed network differed, aligning with gender identity rather than the at birth-assigned sex^41^. Given that transgender persons feel a strong estrangement from their body^48^, which improves after sex hormone treatment^49^, we also hypothesized that the DMN could be less integrated among hormone-naïve transgender persons, and become more integrated with cross-sex hormone treatment. In alignment with expectations, we found that both TrM and TrW had significantly weaker DMN connections than controls, particularly in the mPFC. This observation concurs with our previous findings from a more limited study group of TrM^28^, and supports the notion that TrM and TrW share certain characteristics along the cerebral midline^25^. This is also in line with another previous study of ours investigating cross-sex hormone effects on Cth^44^, where we found that testosterone treatment had similar effects in TrM as estrogen in TrW on the cerebral midline structures that showed greater cortical thickness before treatment. Moreover, the own-body perception among TrW and TrM was less accurate (less congruent with body sex-phenotype of the own unmorphed image) than in controls. The ‘self’ ratings to the 0% morph images increased after treatment, whereas the BM index turned even more negative (greater correspondence of own body with the desired sex) than before treatment.

These new data are at odds with previous reports about different effects of sex hormones on functional connectivity in several different cisgender cohorts. This includes effects among cisgender men and women^39,50–52^. It also contrasts to the reported link between high endogenous testosterone levels and attenuation of resting-state amygdala-prefrontal coupling in adolescents^40^, as well as with the finding that intranasal testosterone reduces amygdala coupling with the orbitofrontal cortex in females^50^. Likewise, it has been shown that healthy users of anabolic steroids (AAS) have reductions in functional connectivity between major hubs for emotional modulation^51^. Together with the present findings these reports raise the question as to whether cis- and transgender persons may react differently to sex hormones, in particular TrM, and at least in the midbrain areas where significant differences between trans and cis persons have been observed. To the best of our knowledge, there are no studies specifically testing this hypothesis. A further possibility is that increase in connectivity in both TrM and TrW leads to a more accurate perception of self. An alternative, and not mutually exclusive, explanation is that androgenization/or estrogenization *of the body* would lead to increased congruence in own-body perception and thus increased functional connectivity. Although the present data do not allow distinction between these two scenarios, the output from the BM test favors the second alternative: cross-sex hormone treatment changed the bodies of our transgender participants making them more congruent with their desired sex. This resulted in higher ‘self’ ratings of the own unmorphed images at the second visit, and a BM index indicating a strengthened trans-gender perception. Thus, it seems less likely (nevertheless, still theoretically possible) that the detected increase of functional connectivity between mPFC and the parietal cortex would represent solely a primary hormone effect on the own body image encoded in the parietal cortex. Future studies that acquire morphometric measurements of multiple body areas that are potentially affected by hormone treatment would help clarify the relationships between hormone treatment, brain connectivity changes, and changes in subjective body congruence. Independently of the exact underlying mechanism, the present data add valuable information to the current literature by providing converging evidence about the involvement of the one's own body perception and self-referential networks in gender dysphoria.

### Methodological considerations

The longitudinal control and transgender groups differed significantly in terms of age, number of years of education, and we did not specifically assess intelligence levels. We, therefore, accounted for age and years of education by adding these as covariates to the baseline group comparisons of functional connectivity. Another potential bias is that the control group consisted of both males and females, as each set was too small individually to conduct meaningful direct sex by group comparisons. However, given that both TrM and TrW differed from controls in a similar manner both before and after treatment, the gender-mixed control group should not be considered as a problem. Further, we did not have reason to believe that effect of time on functional connectivity would be different in male vs. female controls, which would be the primary reason to separate male and female control groups in the present longitudinal study.

We acknowledge that the size and imbalance of control group is a limitation, possibly leading to a general loss of power that would make the likelihood of type-II error (false negatives) higher. We only included subjects with full data sets, which limited the number of controls as the BM test was introduced after we started the inclusion of controls. Notwithstanding, the findings are consistent with several of our previous cross-sectional studies on functional connectivity before hormone treatment; this includes those with larger control groups, and when we have compared male and female controls with TrW and TrM separately, which did not indicate additional regions differing between trans and cis populations (e.g., see^25,28,29^).

One may wonder why we did not carry out two-way ANOVAs when comparing groups and networks, but FSL dual-regression cannot currently be set up this way. As shown in Table 2 we did not have full repetition sets of BM data from controls (full BM data sets were available only for visit 1, scans were, on the other hand, available for both visits. Nevertheless, after submission of this manuscript we carried out full sets of test–retest BM experiments in another group of 10 cisgender controls, showing high test–retest reliability and low (ICC = 0.96). Furthermore, if anything the repetition effect would have led to increased accuracy when evaluating the morphed images, but the effect in our transgender participants was the opposite, reassuring that the observed changes were effects of cross-sex hormone treatment.

Testosterone and estrogen levels were not measured in cisgender controls. None of the cisgender control participants suffered from any sex hormone-related condition, and there were no reasons to assume that their sex hormone values would be abnormal. We did not specifically assess information about the menstrual cycle phase in all the trans men, but the majority were, like the cis women, scanned during the second week of their menstrual cycle.

The interval between scan 1 and scan 2 among cisgender participants was significantly longer compared to the transgender participants. Cisgender participants received no intervention during this time, nor were there any scanner software upgrades or changes in the scanner performance. Nonetheless, in order to further explore the possible effects of the time difference, we carried out post hoc analyses to investigate the interaction between time and brain functional connectivity. Two analyses were carried out, one between TrW and cisgender controls, the other between TrM and cisgender controls for the motor network, salience network and the DMN. No significant cluster emerged in the motor network, which would have been expected if time difference introduced a systematic bias. A significant cluster in the right mPFC was detected in the DMN for the TrM > controls contrast (cluster size = 2.8 cc; maximum 1 − p = 1.0, local maximum x = 4, y = 38, z = 20) and a sub-significant cluster in the corresponding area was detected for the TrW > controls contrast (cluster size = 0.109 cc; maximum 1 − p  = 0.9, local maximum x = 6, y = 44, z = 30), but there were no significant differences in control > TrM or control > TrW. These findings indicate effects above and beyond what occurs with passage of time in controls, inferring that the significant differences can most likely be attributed to hormone treatment in TrM and TrW. From these investigations, it appears unlikely that there was bias associated with the interscan interval (which could equally well show significant differences in control > TrM or control > TrW, which was not the case).

The differential findings in the three networks investigated deserve a comment. Whereas the results in the DMN and salience networks accord with our hypothesis the persistently greater functional connections among controls in the motor networks were unexpected, and need further explanations. In our recent fMRI studies we indeed detected that when judging ‘self’ when viewing own unmorphed bodies cis-gender controls activated sensory motor cortex significantly more than transgender persons^41^. Moreover, our very preliminary data of cortical gyrifications show significantly lower gyrification in the same areas in GD populations. Further investigation of the role of sensory motor cortex in gender identity is obviously warranted.

In conclusion, this study suggests that cross-sex hormone treatment leads to increased perceptual accuracy in own body self-perception, along with increased functional connectivity within the cerebral networks processing this perception, culminating in connectivity patterns in TrW and TrM that are similar to those in cisgender. The similarity of treatment effects among TrM and TrW emphasizes that the neurobiological underpinnings of transgenderism are cerebral midline structures composing parts of the DMN and salience networks.
